# Mortality risks associated with empirical antibiotic activity in *Escherichia coli* bacteraemia: an analysis of electronic health records

**DOI:** 10.1093/jac/dkac189

**Published:** 2022-06-20

**Authors:** Chang Ho Yoon, Sean Bartlett, Nicole Stoesser, Koen B Pouwels, Nicola Jones, Derrick W Crook, Tim E A Peto, A Sarah Walker, David W Eyre

**Affiliations:** Big Data Institute, Nuffield Department of Population Health, University of Oxford, UK; Nuffield Department of Medicine, University of Oxford, UK; Nuffield Department of Medicine, University of Oxford, UK; Nuffield Department of Medicine, University of Oxford, UK; Department of Infectious Diseases and Microbiology, Oxford University Hospitals NHS Foundation Trust, John Radcliffe Hospital, Oxford, UK; The National Institute for Health Research Oxford Biomedical Research Centre, University of Oxford, Oxford, UK; Health Protection Research Unit in Healthcare Associated Infections and Antimicrobial Resistance, University of Oxford, UK; Health Protection Research Unit in Healthcare Associated Infections and Antimicrobial Resistance, University of Oxford, UK; Health Economics Research Centre, Nuffield Department of Population Health, University of Oxford, Oxford, UK; Department of Infectious Diseases and Microbiology, Oxford University Hospitals NHS Foundation Trust, John Radcliffe Hospital, Oxford, UK; Nuffield Department of Medicine, University of Oxford, UK; Department of Infectious Diseases and Microbiology, Oxford University Hospitals NHS Foundation Trust, John Radcliffe Hospital, Oxford, UK; The National Institute for Health Research Oxford Biomedical Research Centre, University of Oxford, Oxford, UK; Health Protection Research Unit in Healthcare Associated Infections and Antimicrobial Resistance, University of Oxford, UK; Nuffield Department of Medicine, University of Oxford, UK; Department of Infectious Diseases and Microbiology, Oxford University Hospitals NHS Foundation Trust, John Radcliffe Hospital, Oxford, UK; The National Institute for Health Research Oxford Biomedical Research Centre, University of Oxford, Oxford, UK; Health Protection Research Unit in Healthcare Associated Infections and Antimicrobial Resistance, University of Oxford, UK; Nuffield Department of Medicine, University of Oxford, UK; The National Institute for Health Research Oxford Biomedical Research Centre, University of Oxford, Oxford, UK; Health Protection Research Unit in Healthcare Associated Infections and Antimicrobial Resistance, University of Oxford, UK; Big Data Institute, Nuffield Department of Population Health, University of Oxford, UK; Department of Infectious Diseases and Microbiology, Oxford University Hospitals NHS Foundation Trust, John Radcliffe Hospital, Oxford, UK; The National Institute for Health Research Oxford Biomedical Research Centre, University of Oxford, Oxford, UK; Health Protection Research Unit in Healthcare Associated Infections and Antimicrobial Resistance, University of Oxford, UK

## Abstract

**Background:**

Reported bacteraemia outcomes following inactive empirical antibiotics (based on *in vitro* testing) are conflicting, potentially reflecting heterogeneity in causative species, MIC breakpoints defining resistance/susceptibility, and times to rescue therapy.

**Methods:**

We investigated adult inpatients with *Escherichia coli* bacteraemia at Oxford University Hospitals, UK, from 4 February 2014 to 30 June 2021 who were receiving empirical amoxicillin/clavulanate with/without other antibiotics. We used Cox regression to analyse 30 day all-cause mortality by *in vitro* amoxicillin/clavulanate susceptibility (activity) using the EUCAST resistance breakpoint (>8/2 mg/L), categorical MIC, and a higher resistance breakpoint (>32/2 mg/L), adjusting for other antibiotic activity and confounders including comorbidities, vital signs and blood tests.

**Results:**

A total of 1720 *E. coli* bacteraemias (1626 patients) were treated with empirical amoxicillin/clavulanate. Thirty-day mortality was 193/1400 (14%) for any active baseline therapy and 52/320 (16%) for inactive baseline therapy (*P *= 0.17). With EUCAST breakpoints, there was no evidence that mortality differed for inactive versus active amoxicillin/clavulanate [adjusted HR (aHR) = 1.27 (95% CI 0.83–1.93); *P *= 0.28], nor of an association with active aminoglycoside (*P *= 0.93) or other active antibiotics (*P *= 0.18). Considering categorical amoxicillin/clavulanate MIC, MICs > 32/2 mg/L were associated with mortality [aHR = 1.85 versus MIC = 2/2 mg/L (95% CI 0.99–3.73); *P *= 0.054]. A higher resistance breakpoint (>32/2 mg/L) was independently associated with higher mortality [aHR = 1.82 (95% CI 1.07–3.10); *P *= 0.027], as were MICs > 32/2 mg/L with active empirical aminoglycosides [aHR = 2.34 (95% CI 1.40–3.89); *P *= 0.001], but not MICs > 32/2 mg/L with active non-aminoglycoside antibiotic(s) [aHR = 0.87 (95% CI 0.40–1.89); *P *= 0.72].

**Conclusions:**

We found no evidence that EUCAST-defined amoxicillin/clavulanate resistance was associated with increased mortality, but a higher resistance breakpoint (MIC > 32/2 mg/L) was. Additional active baseline non-aminoglycoside antibiotics attenuated amoxicillin/clavulanate resistance-associated mortality, but aminoglycosides did not. Granular phenotyping and comparison with clinical outcomes may improve AMR breakpoints.

## Introduction

Antimicrobial resistance (AMR) has received substantial attention for its current and projected threats to safe healthcare worldwide.^1,2^ In high-income countries, Gram-negative bacteria, predominantly *Escherichia coli*, are the leading cause of community-onset bacteraemia, with rising rates of AMR.^3,4^

Timely and effective antibiotic therapy for bacteraemia and sepsis is associated with significant mortality reductions.^5,6^ Empirical therapy, therefore, is typically broad-spectrum, although avoiding unnecessarily broad antibiotic exposure is central to mitigating the spread of AMR and other complications, including *Clostridioides difficile* infection.^7,8^ These competing tensions must be balanced while laboratory antibiotic susceptibility results are awaited. Various terminologies are used to describe the situation where antibiotic(s) given to a patient are resistant on susceptibility testing: discordant, inappropriate, or inactive. We use the term ‘inactive’ throughout, but it should be remembered that this is inactive *in vitro*, rather than necessarily reflecting no activity in patients. Studies investigating outcomes following inactive therapy have shown contrasting results, potentially due to methodological heterogeneity, including different times to definitive rescue antibiotic treatment, inconsistent definitions of discordant/inappropriate/inactive therapy,^9,10^ and different MIC breakpoints defining resistance/susceptibility^11^ (amongst others^5,6^). Some studies include many bacterial taxa,^9,12^ whilst others focus on specific species, often limiting sample sizes.^9,10,13,14^ Two meta-analyses found inappropriate antibiotic therapy was associated with increased mortality in sepsis,^5,6^ whereas studies specifically of Gram-negative bacteraemia observed no overall association.^15,16^

A recent, multicentre US study showed discordant empirical antibiotic therapy for bacteraemia increased in-hospital mortality overall, and varied in frequency across bacterial species (from 5% in β-haemolytic streptococci to 45% in Enterobacterales), as did mortality following discordant therapy, which was highest for *Staphylococcus aureus*, with only moderate evidence of association for Enterobacterales.^12^

Inactive therapy is usually defined using antibiotic susceptibility breakpoints, which are set by expert committees, including EUCAST^17^ and CLSI.^18^ Wild-type distribution of MICs, pharmacokinetic/pharmacodynamic data and clinical outcomes are all considered, but outcome data can be limited.^19^

The advent of 24 h microbiology laboratories (with semi-automated culture/susceptibility testing platforms) and proactive on-call infection consult services potentially reduces the time to effective therapy in those started initially on inactive empirical therapy. This may mitigate any associated harms and influence the necessary breadth of empirical cover. Therefore, we investigated the effect of (in)active, empirical, antibiotic therapy on 30 day mortality following *E. coli* bacteraemia in patients admitted to our hospital group in Oxfordshire, UK, where these service improvements have been in place for several years.

## Methods

We included adults (≥16 years old) with ≥1 blood culture growing *E. coli* during an admission at Oxford University Hospitals, a large UK teaching hospital group, with four hospitals and 1000 beds, serving a population of ∼650 000 and providing specialist referral services.

We included the first positive blood culture per patient per 90 day period and only patients who received amoxicillin/clavulanate (also known as co-amoxiclav, a β-lactam/β-lactamase inhibitor combination of amoxicillin and clavulanate widely used in the UK) within their baseline antibiotic regimen. Amoxicillin/clavulanate was the hospital group’s first-line antibiotic for suspected sepsis, complicated urinary tract infection, moderate/severe community-acquired pneumonia and intra-abdominal infection. Hospital guidelines also recommended additional single-dose gentamicin in patients with sepsis features to cover potential amoxicillin/clavulanate-resistant infections while results were awaited. We also performed a sensitivity analysis, which included each patient only once, considering only the latest bacteraemia episode per patient.

Antibiotic susceptibility was performed using BD Phoenix automated broth microdilution (or disc diffusion when unavailable), following EUCAST guidelines and breakpoints.^17^

Thirty-day all-cause mortality was determined using hospital records that are updated with national data on all deaths.^20^ Follow-up was censored at the earliest of 30 days or the last day the patient was known to be alive from national data or hospital records.

We defined the baseline antibiotic regimen as all IV or oral antibiotics administered in hospital within −12 to +24 h of blood collection for each index *E. coli*-positive culture. Data on antibiotics given in the community prior to admission were not available. We excluded episodes where recorded inpatient antibiotics were commenced >24 h after the index culture.

We considered three models for associations between mortality and *in vitro* amoxicillin/clavulanate activity: (i) using EUCAST breakpoints (resistant >8/2 mg/L); (ii) treating MICs as distinct categories (categorical MIC model); and (iii) a high-level resistance model (MICs > 32/2 versus ≤ 32/2 mg/L), as we found amoxicillin/clavulanate MICs > 32/2 mg/L specifically increased mortality risk in our analysis. We performed an additional sensitivity analysis comparing MICs > 16/2 versus ≤ 16/2 mg/L.

We included two factors to account for other baseline antibiotics: (i) additional active aminoglycosides; and (ii) additional active ‘other’ antibiotics (i.e. neither amoxicillin/clavulanate nor aminoglycoside). We considered aminoglycosides separately as these were typically given as a single additional dose, whereas other antibiotics were generally prescribed for longer. To allow for differing effects of additional antibiotics in patients receiving active or inactive amoxicillin/clavulanate, we used four mutually exclusive categories: (i) active amoxicillin/clavulanate (regardless of other drugs); (ii) inactive amoxicillin/clavulanate alone; (iii) inactive amoxicillin/clavulanate with active aminoglycoside only; and (iv) inactive amoxicillin/clavulanate with ‘other’ active antibiotic. There were insufficient data to include these partial interactions in the categorical MIC model so only main effects for additional antibiotics were included.

We also adjusted for additional baseline factors, including patient characteristics, vital signs, blood tests and hospital factors (see Supplementary methods, available as Supplementary data at *JAC* Online). Index blood cultures taken within the first 48 h of admission were considered community-acquired and those obtained subsequently nosocomial.

We used multivariable Cox regression, accounting for non-linearity of continuous factors, to model time from index blood culture to death within 30 days, including antibiotic exposures irrespective of statistical significance and other factors based on backwards elimination (exit *P *> 0.05; see Supplementary methods).

De-identified data were obtained from the Infections in Oxfordshire Research Database,^21^ which has approvals from the South Central – Oxford C Research Ethics Committee (19/SC/0403), the Health Research Authority and the national Confidentiality Advisory Group (19/CAG/0144).

## Results

**Figure 1.**
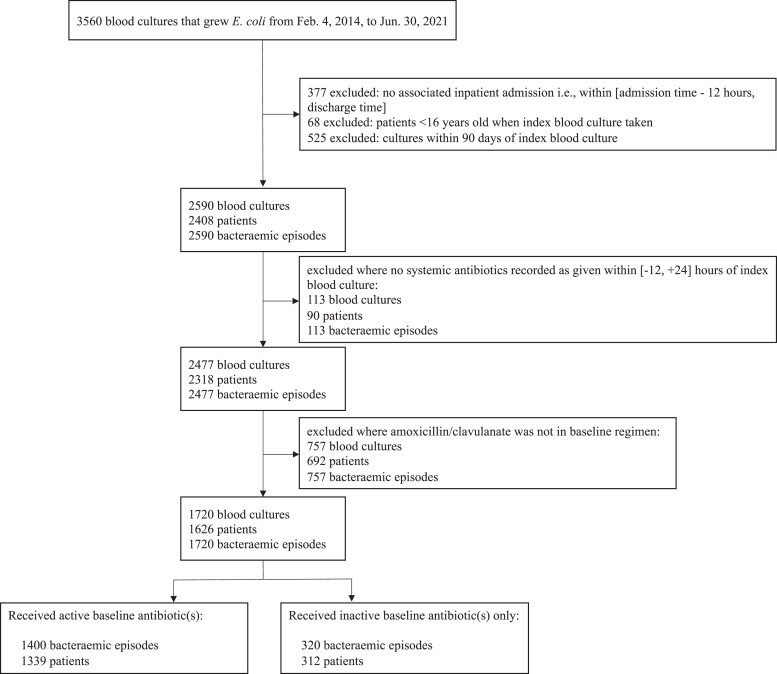
Identification of *E. coli* bloodstream infections included in analysis. Baseline antibiotics comprised those given within −12 to +24 h of blood being taken for the index culture in each bacteraemic episode. Active versus inactive antibiotics were defined by EUCAST breakpoints. Note that 25 patients had multiple infection episodes in which both active and inactive baseline antibiotics were received on different occasions.

Between 4 February 2014 and 30 June 2021, 2590 *E. coli* bacteraemia episodes occurred in 2408 adult inpatients. Excluding episodes with no recorded baseline antibiotics (*n *= 113) or where empirical amoxicillin/clavulanate was not given (*n *= 757) left 1720 episodes in 1626 patients for analysis (Figure 1). By EUCAST breakpoints, 320/1720 episodes (19%) were resistant to amoxicillin/clavulanate, and 74/992 episodes (7%) where a baseline aminoglycoside was given were resistant to aminoglycosides, with resistance to both amoxicillin/clavulanate and aminoglycoside(s) in 65/992 episodes (7%), and resistance to both amoxicillin/clavulanate and another potentially active non-aminoglycoside antibiotic in 209/748 (28%) episodes where both were given.

Overall, 30 day all-cause mortality was 245/1720 (14%), of which 216 (88%) were in-hospital deaths. Of 320 patients, 52 (16%) died in episodes with inactive baseline antibiotics (including amoxicillin/clavulanate), versus 193/1400 (14%) with ≥1 active baseline antibiotic (*P *= 0.17).

Several baseline characteristics differed between episodes with and without active baseline antibiotic(s) (Table 1, Table S1). Prior hospitalization in the last year was more common with inactive baseline antibiotic(s) (57% versus 47% active; *P *< 0.001). Most infections (88% active baseline, 81% inactive baseline; *P *= 0.002) were community onset. ICD10 codes indicated the most commonly documented potential infection sources were urinary [729/1400 (52%) active baseline, 142/320 (44%) inactive baseline; *P *= 0.013] and respiratory [323/1400 (23%) active baseline, 87/320 (27%) inactive baseline; *P *= 0.12]. Multiple potential sources were recorded in 312/1400 (22%) active baseline and 72/320 (22%) inactive baseline cases (*P *= 0.93). Other significant differences between the groups included age at admission [median (IQR) 78 (66–86) years active versus 79 (69–87) inactive; *P *= 0.03], Elixhauser score [median (IQR) 3 (1–4) active versus 3 (2–4) inactive; *P *= 0.04], monocytes [median (IQR) 0.6 × 10^9^ cells/L (0.2–1.0) active versus 0.7 (0.4–1.1) inactive; *P *= 0.001]. Several characteristics also differed across amoxicillin/clavulanate MICs, including highest rates of prior hospitalization and lowest percentage of community-onset infections in cases where the baseline amoxicillin/clavulanate MIC was >32/2 mg/L (Table S2).

**Table 1.** Characteristics of the study population

Characteristic	Active antibiotic(s) in baseline regimen using EUCAST breakpoints (*n *= 1400)	Inactive antibiotic(s) only in baseline regimen using EUCAST breakpoints (*n *= 320)	*P* value
Age, years	78 (66–86)	79 (69–87)	0.032
Sex			0.65
Female	663 (47)	156 (49)	
Ethnicity			0.89
White	1169 (84)	265 (83)	
Other	47 (3)	10 (3)	
Unrecorded	184 (13)	45 (14)	
BMI, kg/m^2^	*n *= 1389	*n *= 287	
	26 (23–29)	25 (22–29)	0.057
Comorbidities			
Diabetes mellitus	358 (26)	83 (26)	0.89
Renal dialysis	15 (1)	3 (1)	1.00
Immunosuppression	177 (13)	37 (12)	0.60
Palliative	29 (2)	10 (3)	0.25
Elixhauser comorbidity score	3 (1–4)	3 (2–4)	0.038
Prior hospitalization^a^	654 (47)	183 (57)	<0.001
Prior episode of *E. coli* bacteraemia^a^	6 (0)	1 (0)	1.00
Specialty			0.36
Acute and general medicine	725 (52)	152 (48)	
Medical subspecialty	308 (22)	77 (24)	
Acute and general surgery	314 (22)	74 (23)	
Other	53 (4)	17 (5)	
Community onset	1225 (88)	259 (81)	0.002
Polymicrobial	155 (11)	35 (11)	0.95
AVPU	*n *= 1227	*n *= 271	0.17
Alert	1139 (93)	258 (95)	
Verbal	74 (6)	9 (3)	
Pain/unresponsive	14 (1)	4 (1)	
Oxygen saturation, %	*n *= 1309	*n *= 287	
	96 (94–98)	96 (94–98)	0.68
Supplementary oxygen	*n *= 1293	*n *= 280	
	382 (30)	77 (28)	0.50
Albumin, g/L	*n *= 1320	*n *= 293	
	29 (25–33)	28 (23–32)	0.038
Alkaline phosphatase, IU/L	*n *= 1318	*n *= 289	
	125 (85–222)	129 (91–229)	0.37
Creatinine, μM	*n *= 1385	*n *= 312	
	98 (74–137)	90 (69–134)	0.070
Urea, mM	*n *= 1385	*n *= 312	
	8 (6–11)	8 (5–12)	0.53
Monocytes, ×10^9^ cells/L	*n *= 1375	*n *= 315	
	0.6 (0.2–1.0)	0.7 (0.4–1.1)	0.001
Neutrophils, ×10^9^ cells/L	*n *= 1375	*n *= 315	
	11 (7–16)	12 (8–16)	0.51
Immature granulocytes, ×10^9^ cells/L	*n *= 1366	*n *= 310	
	0.09 (0.05–0.21)	0.10 (0.05–0.17)	0.85
Deaths within 30 days	193 (14)	52 (16)	0.17
In-hospital	170 (12)	46 (14)	
Out-of-hospital	23 (1.6)	6 (1.9)	

Baseline antibiotics are detailed in Table 2. Data are *n* (%) or median (IQR); *P* values were calculated using Pearson’s chi-squared, Fisher’s exact and Wilcoxon rank-sum tests. Other characteristics including Clinical Classifications Software groups are provided in Table S1, together with univariable HRs for 30 day mortality. Where denominators are not specified, complete data were available.

a Including events up to 1 year before the index blood culture.

Fifty-five of 1400 (4%) active baseline and 2 of 320 (1%) inactive baseline cases received active inpatient antibiotic therapy before the bacteraemia. Metronidazole was co-administered in both active [289/1400 (21%)] and inactive [53/320 (17%)] cases (*P *= 0.099) (Table 2), typically in suspected intra-abdominal infections.

**Table 2.** Antibiotic characteristics

Characteristic	Active antibiotic(s) in baseline regimen using EUCAST breakpoints (*n *= 1400)	Inactive antibiotic(s) only in baseline regimen using EUCAST breakpoints (*n *= 320)	*P* value
EUCAST breakpoints			<0.001
Active baseline amoxicillin/clavulanate	968 (69)	0 (0)	
Inactive baseline amoxicillin/clavulanate only	0 (0)	320 (100)	
Inactive baseline amoxicillin/clavulanate and active aminoglycoside only	266 (19)	0 (0)	
Inactive baseline amoxicillin/clavulanate and active ‘other’	166 (12)	0 (0)	
High-level resistance (MIC > 32 mg/L)	*n *= 1231	*n *= 264	<0.001
MIC ≤ 32 mg/L, baseline amoxicillin/clavulanate	1076 (87)	141 (53)	
MIC > 32 mg/L, baseline amoxicillin/clavulanate only	0 (0)	123 (47)	
MIC > 32 mg/L, baseline amoxicillin/clavulanate and active aminoglycoside only	98 (8)	0 (0)	
MIC > 32 mg/L, baseline amoxicillin/clavulanate and active ‘other’	57 (5)	0 (0)	
Active aminoglycoside	918 (66)	0 (0)	<0.001
Active ‘other’ antibiotic	325 (23)	0 (0)	<0.001
Active pre-culture antibiotics^a^	55 (4)	2 (1)	0.003
Prior hospital exposure to β-lactam antibiotic(s)^b^	443 (32)	136 (42)	<0.001
Antibiotics in baseline regimen (only top eight shown)			
Amoxicillin/clavulanate	1400 (100)	320 (100)	—
Aminoglycoside	944 (67)	48 (15)	—
Metronidazole	289 (21)	53 (17)	—
Ceftriaxone	107 (8)	7 (2)	—
Piperacillin/tazobactam	81 (6)	1 (0)	—
Clarithromycin	60 (4)	21 (7)	—
Ertapenem or meropenem	28 (2)	0 (0)	—
Amoxicillin	23 (2)	8 (2)	—

When considering the activity of the baseline regimen against *E. coli*, only potentially active antibiotics were included, i.e. metronidazole and clarithromycin were excluded. Data are *n* (%) or median (IQR); *P* values were calculated using Pearson’s chi-squared, Fisher’s exact and Wilcoxon rank-sum tests. Baseline antibiotics defined as those received between −12 and +24 h of index blood culture. Where denominators are not specified, complete data were available. EUCAST categorical susceptibility results were available for all cases in the final analysis. For 225 isolates, susceptibility testing was performed by disc diffusion and so MIC results were not available.

a ‘Pre-culture antibiotics’ defined as antibiotics received between −36 and −12 h of index blood culture.

b Includes only in-hospital events up to 1 year before the index blood culture.

**Figure 2.**
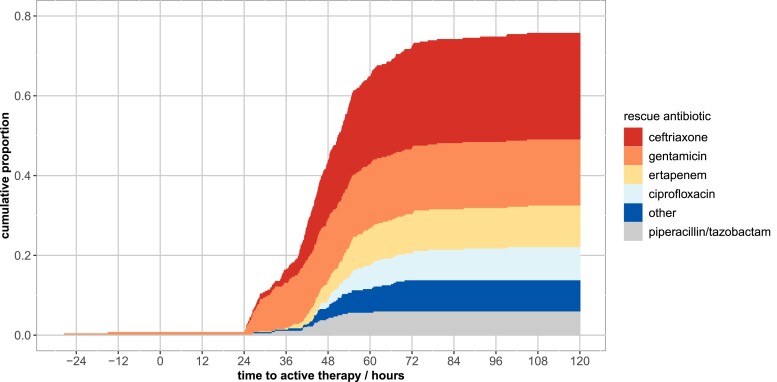
Time to active, rescue antibiotic(s) (h) in the 320 episodes where no active baseline antibiotic therapy was administered. Cumulative proportions are coloured by rescue antibiotic. Two patients received active pre-culture doses of gentamicin at −27 and −15 h prior to index blood culture. Antibiotic activity was based on EUCAST breakpoints.

Of 320 patients, 255 (80%) with inactive baseline antibiotics were recorded to have received active, rescue antibiotic(s): 138/320 (43%) 24–48 h from index blood culture and 230/320 (72%) by 72 h (Figure 2), most commonly ceftriaxone, gentamicin or ertapenem.

### Impact of amoxicillin/clavulanate resistance

**Figure 3.**
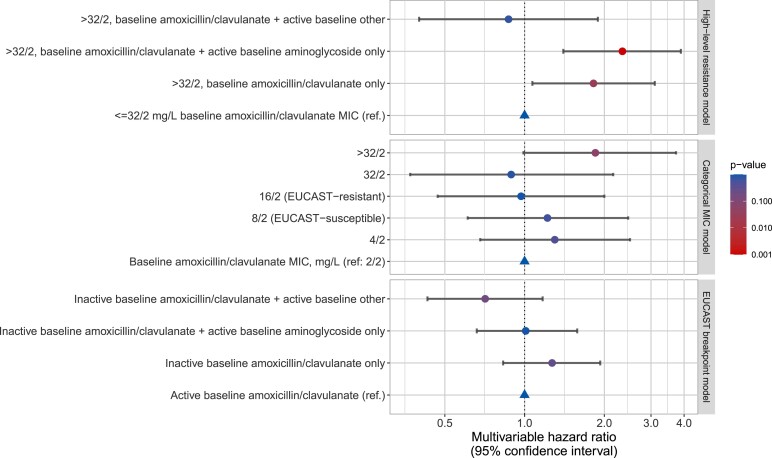
Multivariable relationships between different empirical antibiotic activities and 30 day all-cause mortality in the high-level resistance (>32/2 mg/L), categorical MIC, and EUCAST breakpoint models. For other model covariates see Table 3.

Using EUCAST breakpoints, adjusting for age, BMI, immunosuppression, prior hospitalization, baseline clinical specialty, blood tests and vital signs (Table 3, Figure 3), there was no evidence that 30 day all-cause mortality differed between patients receiving active amoxicillin/clavulanate alone versus inactive amoxicillin/clavulanate alone [adjusted HR (aHR) = 1.27 (95% CI 0.83–1.93; *P *= 0.28)]. Considering categorical amoxicillin/clavulanate MICs, there was moderate evidence that amoxicillin/clavulanate MICs > 32/2 mg/L were independently associated with increased 30 day mortality versus MIC = 2/2 mg/L in both univariable [HR = 1.96 (95% CI 1.06–3.64; *P *= 0.032); Table S3, Figure S2] and multivariable analyses [aHR = 1.85 (95% CI 0.99–3.73; *P *= 0.054)]. There was no evidence of association with MICs of 16/2 or 32/2 mg/L, defined as resistant using EUCAST breakpoints [aHR = 0.97 (95% CI 0.47–2.00; *P *= 0.93) and aHR = 0.89 (95% CI 0.37–2.16; *P *= 0.80), respectively]. Therefore, a third model—the high-level resistance model—compared bacteraemias with amoxicillin/clavulanate MICs ≤ 32/2 versus > 32/2 mg/L. MICs > 32/2 mg/L were independently associated with higher mortality [aHR = 1.82 (95% CI 1.07–3.10; *P *= 0.027)].

**Table 3.** Multivariable Cox regression for 30 day all-cause mortality

	EUCAST breakpoint model (*n *= 1294)		Categorical MIC model (*n *= 1201)		High-level resistance (>32/2 mg/L) model (*n *= 1201)	
	HR (95% CI)	*P* value	HR (95% CI)	*P* value	HR (95% CI)	*P* value
≤32/2 mg/L baseline amoxicillin/clavulanate MIC (ref.)						
>32/2 mg/L, baseline amoxicillin/clavulanate only	—	—	—	—	1.82 (1.07–3.10)	0.027
>32/2 mg/L, baseline amoxicillin/clavulanate + active baseline aminoglycoside only	—	—	—	—	2.34 (1.40–3.89)	0.001
>32/2 mg/L, baseline amoxicillin/clavulanate + active baseline ‘other’	—	—	—	—	0.87 (0.40–1.89)	0.72
Baseline amoxicillin/clavulanate MIC (ref: 2/2 mg/L)						
4/2 mg/L, EUCAST-susceptible	—	—	1.30 (0.68–2.50)	0.42	—	—
8/2 mg/L	—	—	1.22 (0.61–2.46)	0.57	—	—
16/2 mg/L, EUCAST-resistant	—	—	0.97 (0.47–2.00)	0.93	—	—
32/2 mg/L	—	—	0.89 (0.37–2.16)	0.80	—	—
>32/2 mg/L	—	—	1.85 (0.99 –3.73)	0.054	—	—
Active baseline amoxicillin/clavulanate (ref.)						
Inactive baseline amoxicillin/clavulanate only	1.27 (0.83–1.93)	0.28	—	—	—	—
Inactive baseline amoxicillin/clavulanate + active baseline aminoglycoside only	1.01 (0.66–1.58)	0.93	—	—	—	—
Inactive baseline amoxicillin/clavulanate + active baseline ‘other’	0.71 (0.43–1.17)	0.18	—	—	—	—
Active baseline aminoglycoside	—	—	0.88 (0.62–1.24)	0.47	—	—
Active baseline ‘other’	—	—	0.86 (0.57–1.29)	0.46	—	—
Age at admission, 10 years	1.40 (1.20–1.62)	<0.001	1.38 (1.18–1.61)	<0.001	1.39 (1.19–1.62)	<0.001
BMI, kg/m^2^	0.96 (0.92–0.99)	0.019	0.95 (0.92–0.99)	0.0029	0.95 (0.92–0.99)	0.025
Immunosuppression	1.90 (1.31–2.77)	<0.001	2.11 (1.43–3.10)	<0.001	2.03 (1.38–2.99)	<0.001
Prior hospitalization	1.76 (1.23–2.52)	0.002	1.63 (1.13–2.36)	0.009	1.69 (1.17–2.44)	0.005
Specialty (ref: Acute and general medicine)						
Medical subspecialty	0.74 (0.50–1.08)	0.12	0.69 (0.47–1.02)	0.064	0.69 (0.47–1.02)	0.061
Acute and general surgery	0.72 (0.44–1.16)	0.17	0.67 (0.39–1.12)	0.13	0.67 (0.40–1.12)	0.125
Other	0.58 (0.21–1.60)	0.29	0.30 (0.07–1.24)	0.096	0.29 (0.07–1.18)	0.084
AVPU (ref: Alert)						
Verbal	2.12 (1.30–3.46)	0.003	2.25 (1.32–3.82)	0.003	2.16 (1.28–3.64)	0.004
Pain/unresponsive	10.1 (4.45–23.0)	<0.001	6.27 (2.53–15.5)	<0.001	7.69 (3.14–18.9)	<0.001
Supplementary oxygen	2.28 (1.63–3.19)	<0.001	2.23 (1.57–3.17)	<0.001	2.19 (1.55–3.10)	<0.001
Oxygen saturation, %	0.92 (0.87–0.97)	0.003	0.93 (0.87–0.98)	0.008	0.92 (0.87–0.97)	0.003
Albumin, g/L	0.87 (0.84–0.89)	<0.001	0.88 (0.85–0.90)	<0.001	0.87 (0.84–0.90)	<0.001
Alkaline phosphatase, 100 IU/L	1.17 (1.09–1.26)	<0.001	1.17 (1.09–1.26)	<0.001	1.16 (1.08–1.25)	<0.001
Urea, mM	1.06 (1.03–1.08)	<0.001	1.06 (1.04–1.09)	<0.001	1.06 (1.04–1.09)	<0.001
Monocytes, ×10^9^ cells/L	0.61 (0.43–0.87)	0.006	0.56 (0.38–0.80)	0.002	0.57 (0.40–0.82)	0.002
Neutrophils, ×10^9^ cells/L	Figure S1	0.004	similar to Figure S1	0.005	similar to Figure S1	<0.001
Immature granulocytes, ×10^9^ cells/L	1.66 (1.13–2.43)	0.019	1.74 (1.15–2.62)	0.005	1.57 (1.06–2.34)	0.026

Interactions between primary exposures are included in the EUCAST breakpoint and high-level resistance models, but not in the categorical MIC model due to limited power. There were no other significant interactions. AVPU, ‘alert, verbal, pain, unresponsive’.

### Impact of additional active antibiotics

With EUCAST breakpoints, there was no evidence that 30 day all-cause mortality differed between patients receiving active amoxicillin/clavulanate compared with either inactive amoxicillin/clavulanate with active aminoglycoside [aHR = 1.01 (95% CI 0.66–1.58; *P *= 0.93)] or inactive amoxicillin/clavulanate with another active non-aminoglycoside antibiotic [aHR = 0.71 (95% CI 0.43–1.17; *P *= 0.18)] (Table 3, Figure 3).

In the high-level resistance model, adding an active non-aminoglycoside antibiotic in infections with amoxicillin/clavulanate MICs > 32/2 mg/L reduced mortality risk to similar levels as when MICs were ≤32/2 mg/L [aHR versus amoxicillin/clavulanate MIC ≤ 32/2 mg/L = 0.87 (95% CI 0.40–1.89; *P *= 0.72)]. However, amoxicillin/clavulanate MICs > 32/2 mg/L treated with amoxicillin/clavulanate with active aminoglycoside were associated with increased mortality [aHR versus amoxicillin/clavulanate with MIC ≤ 32/2 mg/L = 2.34 (95% CI 1.40–3.89; *P *= 0.001)] (Table 3, Figure 3). We found no evidence that aminoglycosides *per se* increased mortality when given with active amoxicillin/clavulanate [aHR for MIC ≤ 32/2 mg/L with active aminoglycoside versus amoxicillin/clavulanate MIC ≤ 32/2 mg/L without active aminoglycoside = 0.75 (95% CI 0.50–1.11; *P *= 0.15)].

### Other risk factors for mortality

Multiple other factors were independently associated with higher 30 day mortality (Table 3): older age, lower BMI, immunosuppressive condition(s) and prior hospitalization; baseline vital signs, including being unresponsive or responsive only to verbal/painful stimuli, supplementary oxygen; baseline blood test results, including lower albumin, higher alkaline phosphatase, higher urea, lower monocytes, lower neutrophils and higher immature granulocytes; and the baseline clinical specialty (greater mortality associated with acute and general medicine versus medical subspecialty). Higher oxygen saturation was associated with increased mortality, possibly reflecting supplementary oxygen given to the most unwell patients. Since urinary sources were most common, and amoxicillin/clavulanate is predominantly renally excreted, we assessed the impact of additionally adjusting for this in the final high-level amoxicillin/clavulanate resistance model; urinary source was not associated with mortality (*P *= 0.87) and the estimates of effect of amoxicillin/clavulanate MIC and additional antibiotic therapy were similar.

### Sensitivity analyses

In a sensitivity analysis, there was no evidence of an association between mortality and amoxicillin/clavulanate MIC > 16/2 mg/L [aHR versus amoxicillin/clavulanate with MIC ≤ 16/2 mg/L = 1.52 (95% CI 0.93–2.48; *P *= 0.092), Table S4]. Similar results were obtained when restricting our dataset to only include each patient once (Table S5).

## Discussion

In patients with *E. coli* bacteraemia, after accounting for demographics, comorbidities, past hospital exposure and illness acuity, high-level resistance to baseline empirical amoxicillin/clavulanate (MIC > 32/2 mg/L) was associated with increased 30 day mortality. However, we found no evidence that amoxicillin/clavulanate resistance defined using EUCAST breakpoints was associated with mortality (including MICs of 16/2–32/2 mg/L). This disparity between outcomes and antibiotic breakpoints potentially explains some of the heterogeneity in previous studies investigating the impact of AMR in *E. coli* bacteraemia.

Our findings are broadly consistent with a recent US study of 26 036 patients with bacteraemia,^12^ where there was borderline evidence of increased mortality in patients with Enterobacterales bacteraemia who received inactive empirical antibiotic therapy [OR = 1.23 (95% CI 1.00–1.52; *P *= 0.054)]. However, this study did not examine the impact of MIC. Another study of Enterobacterales bacteraemia highlighted the methodological differences between CLSI and EUCAST for determining amoxicillin/clavulanate MICs, and found no association between MIC and mortality, by either method and using a variety of breakpoints, but had only limited power with 202 *E. coli* cases.^11^

In addition to highlighting adverse outcomes from AMR, our findings suggest that breakpoints for amoxicillin/clavulanate may be set too conservatively, at least for some bloodstream infections. Interestingly, the EUCAST amoxicillin/clavulanate breakpoint for Enterobacterales causing uncomplicated urinary tract infection is >32/2 mg/L, i.e. the level of resistance we found was associated with increased mortality. As urinary tract infection was the most common, presumed bacteraemia source in our study, it is possible that urinary excretion of amoxicillin/clavulanate, coupled with high blood concentrations from IV administration, were sufficient to treat infections with MICs of 16/2–32/2 mg/L. Our approach highlights more generally the benefit of large-scale electronic health records as a tool for reviewing and setting antibiotic breakpoints, with a greater focus on patient outcomes than has previously been possible.

Additional active baseline empirical non-aminoglycoside antibiotics, predominantly cephalosporins and carbapenems, negated the impact of high-level amoxicillin/clavulanate resistance. These additional antibiotics were started prior to microbiology results becoming available, typically replacing amoxicillin/clavulanate, for example, following senior medical review, or transfer from the emergency department to the admitting speciality. There was evidence that these antibiotics as a group were beneficial, i.e. we did not assess variation between cephalosporins versus carbapenems.

In contrast, higher mortality with high-level amoxicillin/clavulanate resistance was similar whether additional active aminoglycoside (majority single-dose gentamicin) was given or not. As local guidelines recommended single-dose gentamicin in patients with high-risk sepsis features, this may simply be a marker that patients who received gentamicin were more unwell. However, we adjusted for illness severity using vital signs and laboratory tests, meaning this is unlikely to be the full explanation. Another possible explanation is that local guidelines recommended gentamicin doses of 3–5 mg/kg to minimize toxicity, in contrast to higher doses, ≥7 mg/kg, that may be required to achieve adequate peak concentrations, particularly among critically ill patients.^22,23^ However, our findings may indicate that single-dose aminoglycoside is insufficient to rescue patients with *E. coli* bacteraemia highly resistant to amoxicillin/clavulanate. Data on the clinical impact of one-off aminoglycosides in *E. coli* sepsis is sparse, although support exists for its use in less severe infections, e.g. cystitis.^24^ In general, combining aminoglycosides with a β-lactam in sepsis does not reduce mortality, but does increase nephrotoxicity.^25^ Similarly, whilst aminoglycosides are widely used as an adjunct in neutropenic sepsis with an antipseudomonal β-lactam, this is not backed by guidelines or studies.^25,26^

A study strength is that we adjust for confounding more completely than in many other studies, e.g. we account for prior healthcare exposure and other factors that increase the risk of AMR and may also be associated with adverse outcomes. We also used both vital signs and laboratory tests to ensure differences in illness severity at initial presentation were robustly accounted for. Consequently, we found multiple other independent associations with increased mortality: greater age, lower BMI (potentially reflecting lower physiological reserve), prior hospitalization and the presence of immunosuppression, consistent with existing evidence.^9,12,27–31^ Haematology and biochemistry tests, unavailable to the same extent in other recent studies,^9,11,12,32^ were strongly associated with mortality. Hypoalbuminaemia, raised alkaline phosphatase and uraemia were associated with increased mortality.^33–37^ Neutropenia can be a risk factor or a consequence of sepsis; in this cohort, as amoxicillin/clavulanate was chosen as the primary antibiotic, patients were not believed *a priori* to have neutropenic sepsis (as local guidelines recommended piperacillin/tazobactam for this); therefore, most of the observed neutropenia, associated with increased mortality, is likely to be a consequence of acute infection. Low monocytes were associated with increased mortality, as previously described.^38^ Associations between specific white cell lineages and infection outcomes are also seen in other infections, e.g. eosinopenia and *C. difficile*,^39^ and basophils and eosinophils in COVID-19.^40^ Amongst vital parameters, alertness level, oxygen saturation and the recorded use of supplementary oxygen were also associated with mortality.

Other study strengths include its focus on the impact of AMR in *E. coli* bacteraemia, the most common Gram-negative pathogen, thereby highlighting organism-specific associations with mortality; this study is also one of the largest to assess the clinical impact of EUCAST breakpoints versus alternative definitions of ‘active’ empirical antibiotic therapy for this pathology. We mitigated some limitations of previous studies: heterogeneity of empirical antibiotic choice by selecting patients administered at least baseline amoxicillin/clavulanate; ambiguity of ‘inappropriate’ therapy by using *in vitro* susceptibility-based definitions to define ‘active’ versus ‘inactive’ therapy; and assessing the MIC continuum of susceptibility.

Limitations include the lack of data on community-prescribed antibiotics prior to hospital admission, in particular because AMR potentially contributes to failure of community treatment, need for hospitalization and illness severity at admission. Since some of our model variables (e.g. vital signs, blood tests) capture illness severity at admission, we may have underestimated the overall association between MIC and mortality by adjusting for factors that may mediate the pre-hospital impact of AMR. There were relatively fewer patients with MICs of 32/2 mg/L, which limited the power of our study to detect differences in mortality at this MIC; however, point estimates from the categorical model suggested that it was MICs > 32/2 mg/L specifically that were associated with increased mortality rather than 16/2 and 32/2 mg/L. We did not further quantify MICs > 32/2 mg/L, given the limits of the assay used. This was a study of electronic health records from a single hospital group serving a relatively ethnically homogeneous population, with implications for generalizability. Another limitation is that the study antibiotic is a combination of two drugs (amoxicillin and clavulanate) with variation between EUCAST and CLSI approaches to susceptibility testing, with the former using a fixed clavulanate concentration and the latter a fixed ratio, which has implications for which isolates are reported as resistant.^41^ Furthermore, in the UK, amoxicillin/clavulanate is the leading β-lactam/β-lactamase inhibitor with an amino-penicillin in use, with a maximum dose of 3 g of amoxicillin component per 24 h. Underdosing of the β-lactam component is another possibility to account for higher mortality seen. A similar β-lactam/β-lactamase inhibitor is ampicillin/sulbactam, which is not marketed in the UK, but can be given in doses of up to 8 g for the ampicillin component in 24 h.

Further work should aim to assess the clinical impact of more granular AMR phenotypes in other causes of sepsis, including other Enterobacterales species, whilst adjusting for severity of illness, capturing both chronic and acute patient contexts. It may also be that bacterial genotypes, i.e. specific resistance mechanisms, are also important in determining outcomes.^42^ Identifying which patients are most at risk of inactive empirical treatment, hence most at risk of adverse outcomes, could potentially improve patient outcomes.

In summary, high-level (>32/2 mg/L) amoxicillin/clavulanate resistance is associated with increased mortality from *E. coli* bacteraemia. Disentangling the heterogeneous impact of AMR on mortality may require an organism-specific approach. High-quality electronic healthcare record studies, coupled with more granular resistance phenotyping and genotyping, may improve antibiotic resistance breakpoint setting and potentially in the future lead to clinical guidelines based on MICs and specific patient factors, which in turn may improve outcomes for patients.

## Supplementary Material

dkac189_Supplementary_Data

## Data Availability

The data analysed are not publicly available, as they contain personal data, but are available from the Infections in Oxfordshire Research Database (https://oxfordbrc.nihr.ac.uk/research-themes-overview/antimicrobial-resistance-and-modernising-microbiology/infections-in-oxfordshire-research-database-iord/), subject to an application and research proposal meeting on the ethical and governance requirements of the Database.

## Supplementary data

Supplementary methods, Tables S1 to S5 and Figures S1 to S3 are available as Supplementary data at *JAC* Online.
